# Structural and Biochemical Aspects Related to Resistance and Susceptibility of Rubber Tree Clones to Anthracnose

**DOI:** 10.3390/plants10050985

**Published:** 2021-05-14

**Authors:** Izabela Ponso Magalhaes, João Paulo Rodrigues Marques, Marcela Eloi Gomes, Erivaldo José Scaloppi Junior, Ivan Herman Fischer, Edson Luiz Furtado, Rodney Lucio Pinheiro Henrique, Flavia Thomaz Veréchia Rodrigues, Ana Carolina Firmino

**Affiliations:** 1 College of Agricultural and Technological Sciences, São Paulo State University (Unesp), Dracena 17900-000, São Paulo State, Brazil; marcelaeloi14g@gmail.com (M.E.G.); rodney.lucioh@gmail.com (R.L.P.H.); flavia.verechia@unesp.br (F.T.V.R.); 2 Center for Nuclear Energy in Agriculture, University of São Paulo (USP), Piracicaba 13400-970, São Paulo State, Brazil; joaoanatomia@gmail.com; 3 Center of Rubber Tree and Agroforestry Systems, Agronomic Institute of Campinas (IAC), Votuporanga 15505-970, São Paulo State, Brazil; scaloppijr@yahoo.com.br; 4 Central-West Regional Center, São Paulo’s Agency for Agribusiness Technology (APTA), Bauru 17030-000, São Paulo State, Brazil; ihfische@gmail.com; 5 School of Agriculture, São Paulo State University (Unesp), Botucatu 18610-034, São Paulo State, Brazil; edson.furtado@unesp.br

**Keywords:** *Colletotrichum*, *Hevea brasiliensis*, lignins, lipids, proteins, stomata

## Abstract

The aim of the present study was to evaluate structural and biochemical aspects related to the interaction of resistant (RRIM 937, IAC 502 and 507) and susceptible (RRIM 600) rubber tree clones with *C. tamarillo*. For such analysis, ultrathin sections of the leaf limb were embedded in historesin and differently stained to verify structural alterations and presence of starch grains, arginine, lipids, tannins and lignins. The total proteins and activity of the enzymes peroxidase and (PAL) were quantified. Stomatal density was also analyzed under a scanning electron microscope. Data indicated alterations in the cell content of resistant clones inoculated with the pathogen, as well as greater lignin and lipid accumulation in these samples. For tannins, there was no difference between inoculated and non-inoculated clones. Arginine was found at greater quantities in IAC 502 and 507. Starch grains were not detected in any of the analyzed samples. Protein level and stomatal density were lower in resistant clones. Peroxidase activity was more expressive in resistant clones. PAL activity, there was no significant difference between clones. The lignin and lipids, total protein, peroxidase activity and stomatal density may be related to the resistance of rubber tree clones to anthracnose.

## 1. Introduction

The use of resistant materials is considered ideal to control diseases since they can be applied to large areas and have low environmental impact. Plant resistance to pathogens can be classified as qualitative and quantitative [1,2]. There is evidence that resistance of rubber trees to anthracnose, caused by fungi belonging to the genus *Colletotrichum*, is qualitative since different resistance degrees have been reported among the tested clones. A recent study developed by Antonio et al. [3], in the region of Votuporanga City, São Paulo State, Brazil, identified different disease severity levels among 22 clones tested in the field, and the most resistant clones were IAC507 and IAC505, followed by IAC502, RRIM937, PB235 and PB311, which presented moderate symptoms. In that study, the clone RRIM600 showed the highest susceptibility to anthracnose, both in the field and in the laboratory. 

Similar results were obtained by Qianchun [4] for other genotypes in China, both in the field and in the nursery, using 34 clones from 1984 to 1989. Based on the development of infection by *C. gloeosporioides* in different rubber tree clones, that author reported longer latent period and shorter sporogenesis resulting in lower disease severity for resistant clones, in contrast to shorter incubation period, earlier sporogenesis and greater disease severity for susceptible clones [4]. Magalhães et al. [5], noticed those same aspects for *C. tamarillo*-rubber tree pathosystem of resistant (IAC 502, IAC 507 and RRIM 937) and susceptible (RRIM 600) plants.

The mechanisms involved in the plant resistance to pathogens can be divided into pre-formed mechanisms, which are present in the plant before its contact with a potential pathogen, and post-formed mechanisms, which are activated in response to the presence of a pathogen. They are subdivided into structural and biochemical mechanisms. Structural mechanisms consist in barriers imposed by the plant anatomy, preventing the pathogen from entering or colonizing tissues. Stomata (number, morphology and opening period) and cuticle (composition and thickness), and cytoplasmic aggregations and cell lignification are examples of pre and post-formed structural mechanisms, respectively [6]. Biochemical defense mechanisms consist in substances that are toxic or provide adverse conditions for the pathogen development inside the host. This category includes production of peroxidase and phenylalanine ammonia lyase (PAL). Such enzymes can impair the establishment of the disease since they interfere with the entrance of the pathogen [6]. Rubber trees have been reported to present accumulation of some substances like peroxidase, scopoletin and phenolic compounds when attacked by *Phytophthora palmivora* [7].

Studies related to cytological observations and enzyme production related to the development of a microorganism and the plant cell response can provide significant information for physiological and molecular investigations regarding compatibility or incompatibility in plant-host interactions [6]. The events that determine host resistance or susceptibility often occur in a limited population of cells or in cells close to the host pathogen interface in the area known as the infection site. Thus, cytological approaches are still of great importance in the analysis of biological phenomena and in the understanding of plant-host interaction [8].

Thus, the present study aimed to evaluate structural (stomatal density) and biochemical (total proteins, lignins, tannins, starch grain, arginine, lipids and enzymes) resistance mechanisms that may be involved in the interaction of rubber trees with *C. tamarillo*, complementing the understanding of this pathosystem.

## 2. Material and Methods

### 2.1. Inoculation and Preparation of Leaflets from Rubber Trees for Histological Analysis

The leaflets employed in the experiments were collected from the middle part of clones RRIM 600 (susceptible to anthracnose) and IAC 507, IAC 502 and RRIM 937 (resistant to anthracnose). The plants were six years old, on average, and were grown in a clonal garden of São Paulo’s Agency for Agribusiness Technology (APTA), Center of Rubber Tree and Agroforestry Systems, Agronomic Institute of Campinas (IAC), Votuporanga, São Paulo State, Brazil.

The study was developed with a *C. tamarillo* isolate from rubber tree, stored at the Mycology Collection of Forest Pathology of São Paulo State University (Unesp), College of Agricultural and Technological Sciences, Dracena, São Paulo State, Brazil, which was molecularly identified through sequencing of part of its DNA (MW031267). To obtain the inoculum for the experiments, the fungus was cultured in oat medium, at 25 ± 1 °C and continuous photoperiod, for seven days. The colonies were washed in sterile distilled water and the obtained suspension was filtered in sterile gauze, quantified in a Neubauer chamber, and adjusted to 10^5^ conidia/mL.

Leaflets from rubber tree clones RRIM 600, RRIM 937, IAC 502 and IAC 507 were disinfected superficially with NaClO at 2% and washed in sterile water; then, they were inoculated with 30µL aliquots containing conidial suspension (10^5^ conidia/mL) of the isolate. The inoculated area was delimited with plastic adhesives. Leaflets treated only with a 30 µL aliquot of sterile water, without the presence of the fungus, were also used for comparison with the inoculated samples.

Following inoculation, the leaflets were kept in a humid chamber, at 30 ± 1 °C, in the dark, under high saturation and humidity, for 24 h. Subsequently, 5mm-diameter samples were obtained from the inoculated areas and fixed in “Karnovsky” solution (2.5% glutaraldehyde, 2.0% paraformaldehyde, 0.05 M phosphate buffer, pH 7.2). After 24 h, the samples were removed from the fixative and processed according to the methodology described by Firmino et al. [9].

The sections were stained with toluidine blue, which is classified as a metachromatic dye for showing a different coloration according to its reaction to the substrate. Mucilage and pectin-rich walls will turn purple, cellulosic walls will become blue, and lignified walls and non-structural phenolic compounds may turn green or greenish-blue [10,11]. To visualize starch grains, arginine, total lipids and tannins, the histological sections were stained with Lugol, α-Naphthol, Sudan III and vanillin-hydrochloric acid, respectively [10,11]. For visualization of lignin, which is known to have natural fluorescence, unstained sections were analyzed under an ultraviolet light optical microscope, using 490nm excitation filter [12,13].

Slides containing the processed samples were analyzed under a Jena Lumar microscope, Zeiss, located at School of Agriculture, São Paulo State University (Unesp), Brazil, São Paulo State, Botucatu. An Opton video-camera system, model TA-0124XS, coupled to an optical microscope, was used to capture the images, which were compared among clones. The evaluation of this experiment was of the qualitative type, observing and comparing the presence and understanding of the compounds in the different treatments.

### 2.2. Quantification of Total Proteins in Rubber Tree Clones

To quantify protein levels, protein extract was obtained from the leaves of all 4 studied clones. Five leaves were randomly collected from the middle sprouting part of three plants of each clone, totaling 15 leaves for each studied clone. The collected leaves did not show symptoms of the disease.

From each collected leaf, 0.5g fresh sample was weighed, ground in liquid nitrogen and mixed with 2 mL sodium acetate buffer (0.1 M and pH 5.0) containing 1.0 mM EDTA and 0.3 g polyvinylpyrrolidone (PVP). Then, these sample were kept at −20 °C in a freezer and, after 12 h, centrifuged at 12000 rpm, for 30 min, at 4 °C; the supernatant was transferred to new 1.5 mL tubes, and the samples were stored at −80 °C. Each weighed fresh sample generated two 1.5 mL tubes of supernatant, both of which were analyzed as replicates for the readings of each evaluated sample. To quantify total protein level, the Bradford method was adopted [14].

### 2.3. Activity of the Enzymes Peroxidase and PAL in Different Rubber Tree Clones

For these analyses, the clones had leaves (with symptoms and without symptoms) collected from their apical part, which shows predominance of the disease and is exposed to the pathogen under natural field conditions, since anthracnose is already known to naturally occur in the studied clonal garden. Five leaves were collected from each of three trees in the clonal garden.

The activity of peroxidase was analyzed according to the methodology described by Hammerschimidt et al. [15], while the activity of PAL was determined through colorimetric quantification of trans-cinnamic acid released from phenylalanine substrate, based on the methodology described by Umesha [16]. All enzymatic assays were performed in duplicate for each treatment.

### 2.4. Quantification of Stomata under Scanning Electron Microscope

To complement the study, the quantity of stomata in the clones was obtained under a scanning electron microscope. First, leaf fragments (around 5mm diameter) were collected from each clone and fixed in “Karnovsky” (2.5% glutaraldehyde, 2.0% paraformaldehyde, 0.05 M phosphate buffer, pH 7.2) for a minimal 24 h period. Then, they were processed, according to the methodology described by Firmino et al. [9], for analysis under a LEO435-VP scanning electron microscope, located at the Center for the Electron Microscope of Luiz de Queiroz College of Agriculture (ESALQ), University of São Paulo (USP), Piracicaba, São Paulo State, Brazil. The obtained images were used to determine the number of stomata on the abaxial leaf surface. The evaluation included 15 leaves, which were collected from different plants of one same clone (five leaves per plant). From each leaf, 10 images were captured for counting the stomata.

### 2.5. Data Analysis

The histological evaluation of this experiment was of the qualitative type, observing and comparing the presence of the compounds in the different treatments.

The total proteins, activity of the peroxidase and PAL enzymes and number of stomata were statistically analyzed by comparing the averages of the values received according to the Tukey test, at 5% probability, using the SISVAR software, developed by UFLA - Federal University of Lavras, Paraná, Brazil. [17].

## 3. Results and Discussion

### 3.1. Histological Analysis of Leaflets from Rubber Trees

**Figure 1:**
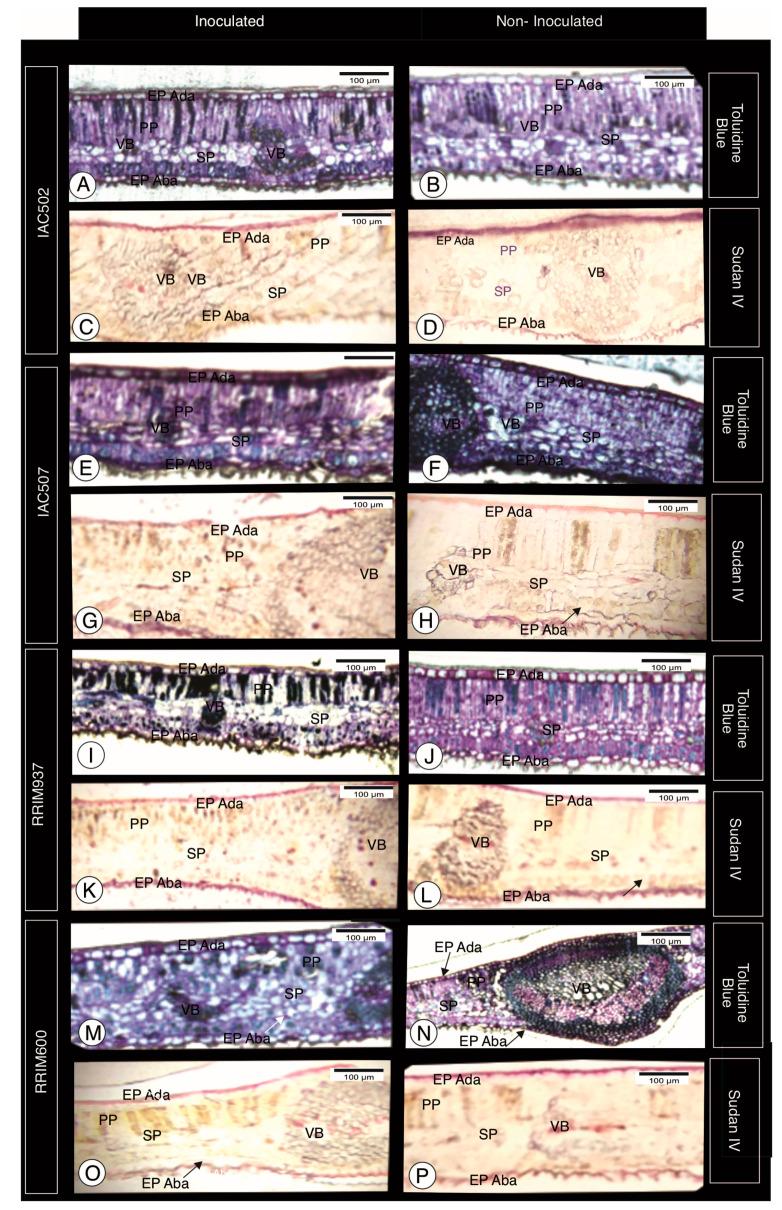
Histological sections stained with toluidine blue and Sudan IV, for lignin visualization, from clones IAC 502 (**A**–**D**), IAC 507 (**E**–**H**), RRIM 937 (**I**–**L**) and RRIM 600 (**M**–**P**). Inoculated (**left**) and non-inoculated clones (**right**). EPI Ada—Epidermis Adaxial; EPI Aba—Epidemis Abaxial; PP—Palisade Parenchyma; SP—Spongy Parenchyma; VB—Vascular bundle.

Changes were detected in the epidermis and palisade parenchyma of clones inoculated with *C. tamarillo*, which may be justified by mucilage or pectin accumulation (Figure 1). Such granular content may correspond to a structural response of the plant after the pathogen attack and can be related to cytoplasmic aggregation, since these aggregates have cell structures like rough endoplasmic reticulum and Golgi apparatus associated with normal biosynthesis processes and possibly secrete materials that can be used in the formation of halos and papillae [18]. However, more specific histochemical tests are needed to visualize such reactions.

Both susceptible and resistant plants have large quantities of lipids in their epidermis, but even greater quantities were observed in inoculated plants, especially in the spongy parenchyma (Figure 1). Lipids and their metabolites can interference the plant-pathogen interactions, including in the resistance mechanisms of the plant and in the pathogenesis stage. Some microorganisms can detect the presence of lipids in a host, mainly those present in the plant surface wax [6,19,20]. They can produce toxins that have the plant lipid metabolism as target. In contrast, plants evolved to recognize microbial lipopolysaccharides (LPSs), sphingolipids and lipid-biding proteins as elicitors of defense response. Recent studies have demonstrated that membrane lipids interact resistance proteins that recognize pathogen-derived effectors, developing specific resistance [19,20]. The plant cell membranes also serve as reservoirs for the release of biologically active, such as jasmonic acid, which is involved in signaling and modulation of plant defense responses [19]. During a stress response, lipids of the fatty acid type tend to be peroxidized by lipoxygenase or reactive oxygen species, originating metabolites involved in the host defense [20].

Starch grains were not found in any of the analyzed samples. As starch is synthesized in the leaves during the day, from carbon fixed by photosynthesis, and mobilized during the night to assist in continuous respiration and saccharose exportation, possible reasons for not observing it were the short evaluation of the infection, the adopted inoculation method (detached leaves), or the time of leaf collection. In addition, it must be considered that this substance is accumulated in storage organs, including seeds, fruits, tubers and storage roots [21].

**Figure 2:**
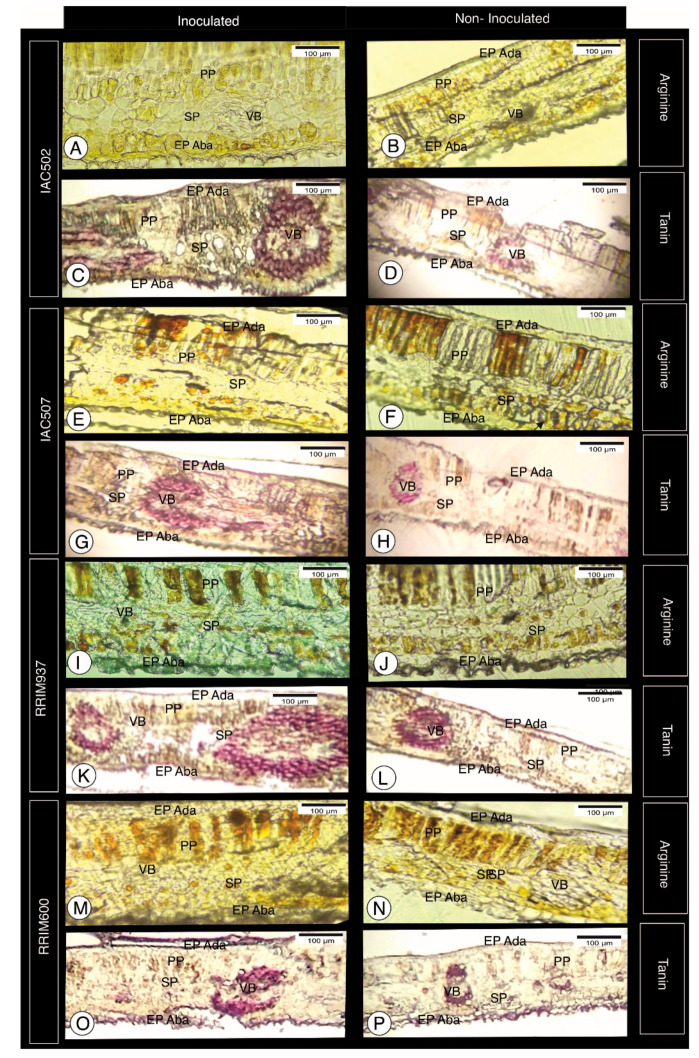
Histological sections stained with α-Naphthol, for arginine visualization (yellow), and vanillin-hydrochloric acid, for tannin visualization (pink), from clones IAC 502 (**A**–**D**), IAC 507 (**E**–**H**), RRIM 937 (**I**–**L**) and RRIM 600 (**M**–**P**). Inoculated (**left**) and non-inoculated clones (**right**). EPI Ada—Epidermis Adaxial; EPI Aba—Epidemis Abaxial; PP—Palisade Parenchyma; SP—Spongy Parenchyma; VB—Vascular bundle.

Arginine was found at greater quantities near the epidermis close to the stomata, and clones IAC 502 and IAC 507 seemed to accumulate more of this group of protein amino acids, whether they were inoculated or not (Figure 2). Together with serin, arginine forms a complex that influences the constitutive and alternative processes of plant defense against biotic and abiotic agents [22]. This characteristic corroborates the present results since those two clones were more resistant than RRIM 937, intermediately resistant, and RRIM 600, susceptible to anthracnose [3]. Although several steps of its biosynthesis remain poorly characterized in plants, arginine is known to be a precursor of nitric oxide. In contrast, conversion of arginine into polyamines is well documented, and several plant species may also have ornithine as a precursor of polyamines. Both nitric oxide and polyamines play essential roles in the regulation of plant development processes, as well as in the responses to biotic and abiotic stress. Therefore, arginine catabolism may serve not only to mobilize nitrogen sources but also to adjust the plant development and defense mechanisms against stress, including attacks of microorganisms [23].

Tannins constitute a group of phenolic polymers with defense properties against microorganisms [24]. In the analyzed samples, they were found at greater quantities in conductive vessels and at low quantities in the epidermis, slightly differing between inoculated and non-inoculated plants (Figure 2). According to Scalbert [25], the toxicity of tannin to fungi, bacteria and yeasts can be explained by different mechanisms, such as inhibition of extracellular microbial enzymes, suppression of substrates necessary for microbial growth or direct action on the microbial metabolism through oxidative phosphorylation inhibition. However, several microorganisms are known to be capable of surpassing the tannin-based defenses of plants. They can process such substances through polymer synthesis, oxidation, tannin biodegradation or synthesis of siderophores.

**Figure 3:**
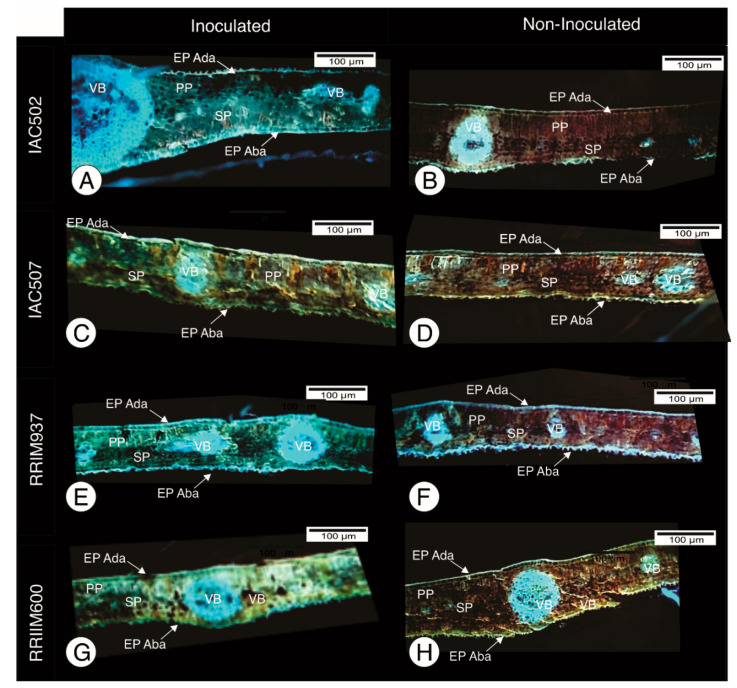
Histological sections under UV light, for lignin visualization (blue fluorescence), from clones IAC 502 (**A**,**B**), IAC 507 (**C**,**D**), RRIM 937 (**E**,**F**) and RRIM 600 (**G**,**H**). pl: spongy parenchyma pp: palisade parenchyma; Ep ada: abaxial epidermis. Bar=200µm. Inoculated (**left**) and non-inoculated clones (**right**). EPI Ada—Epidermis Adaxial; EPI Aba—Epidemis Abaxial; PP—Palisade Parenchyma; SP—Spongy Parenchyma; VB—Vascular bundle.

The presence of lignin was more significant in the inoculated plants than in the non-inoculated ones (Figure 3), which indicates that it can be one of the main defense mechanisms of rubber tree against anthracnose. Cell wall thickening and lignin accu-mulation in the epidermis and vessels were observed. Autofluorescence occurs for plant-pathogen combinations showing a hypersensitivity response originated from the incompatibility between the pathogen and the plant, i.e., certain plant resistance [12]. First, the cells may use decompartmentation to activate the rapid oxidation of their phenolic content and the consequent lignification and suberization of cells, causing their death, in order to interfere with the infection processes or injuries at the immediate site of cell penetration; secondly, if such defense fails and the stress persists, those same processes pro-mote prolonged accumulation of indole acetic acid and ethylene, which cause an additional metabolic cascade in peripheral cells, including secondary metabolism and growth responses to produce peridermic defense in depth [26].

### 3.2. Quantification of Total Proteins in Rubber Tree Clones

**Figure 4:**
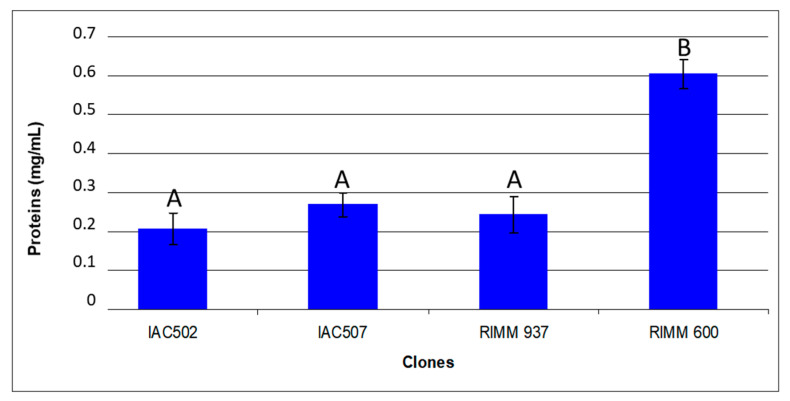
Average protein values in the tested rubber tree clones (IAC502, IAC 507, RRIM 937 and RRIM 600). Average values followed by the same letter do not differ, according to Tukey’s test, at 5% probability. Coefficient of variation: 32.05.

The total protein level was greater in clone RRIM 600 than in the remaining resistant clones (Figure 4), which may be associated with the plant resistance/susceptibility reaction since some enzymes produced by the pathogen, such as proteases, are part of the signaling necessary for the infectious process [1,6]. Redman and Rodriguez [27] developed a mutant of *C. coccodes* that did not produce protease, showing the importance of this enzyme for the fungus in the infectious process, since the mutants could not cause disease in tomato plants. Thus, a higher protein level in susceptible plants may imply better substrate recognition by the pathogen for the establishment of parasitic relationships. However, it must be highlighted that the protein level may vary according to several factors like the age of the leaf and the nutritional conditions of the plant.

**Figure 5:**
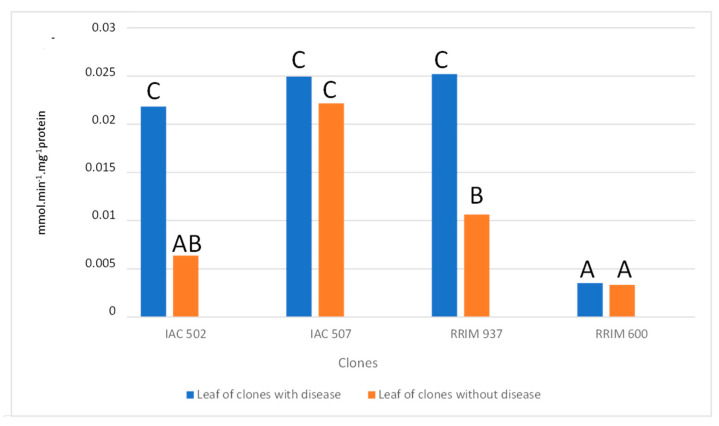
Activity of the enzymes peroxidase in the leaves of rubber trees with and without the presence of anthracnose. Average values followed by the same letter do not differ, according to Tukey’s test, at 5% probability. (Tukey’s test; *p* < 0.05, Coefficient of variation: 31.57).

**Figure 6:**
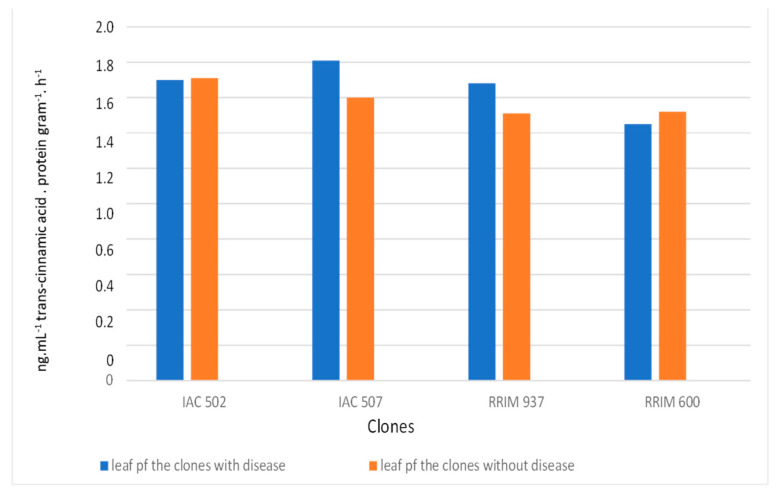
Activity of the enzymes PAL in the leaves of rubber trees with and without the presence of anthracnose. Non-significant difference (5% F test).

The activity of peroxidase was more expressive in resistant clones, especially in samples that showed symptoms of anthracnose (Figure 5). Such results corroborate the data obtained in the analyses for presence of lignin. Peroxidase catalyzes the oxidation and the eventual polymerization of hydroxycinnamic alcohol groups in the presence of hydrogen peroxide, forming lignin; it also participates in the oxidation of phenolic compounds, which accumulate as a response to infection [6,28]. Although PAL is related to different phenolic compounds of plant defense, which are present in the formation of esters, flavonoids and lignins [6], its activity had no significant difference between treatments (Figure 6).

### 3.3. Quantification of Stomata under Scanning Electron Microscope

**Figure 7:**
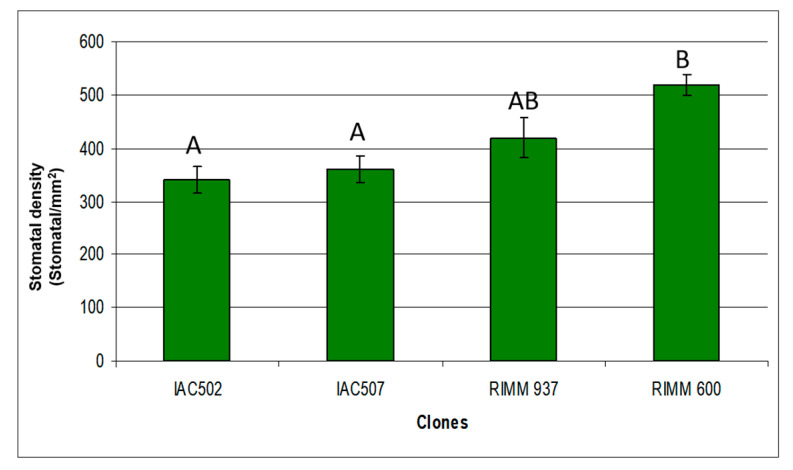
Average density of rubber tree clones (IAC502, IAC 507, RRIM 937 and RRIM 600). Means followed by the same letter do not differ, according to Tukey’s test, at 5% probability. Coefficient of variation: 15.80.

**Figure 8:**
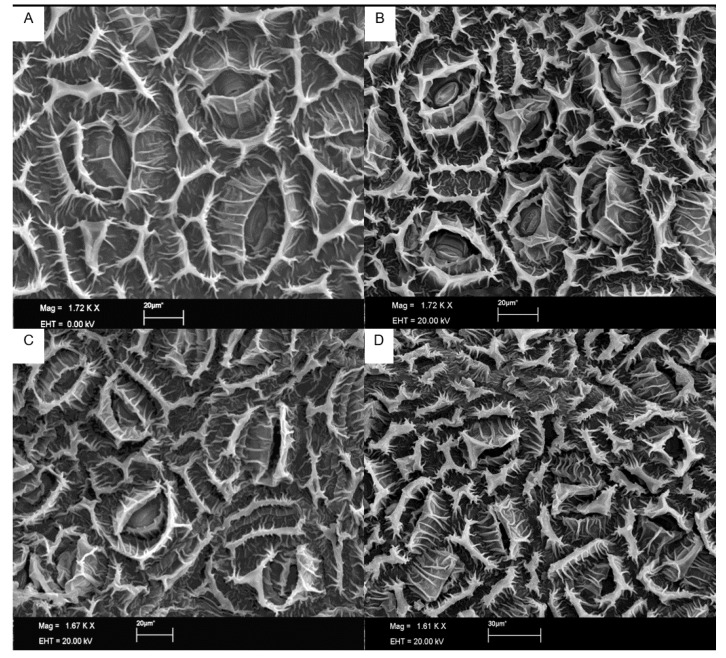
Scanning electron microscopy photos of stomata in the leaves of rubber tree clones IAC 502 (**A**), IAC 507 (**B**), RRIM 937 (**C**) and RRIM 600 (**D**).

Stomatal density was lower in clones IAC 502, IAC 507 and RRIM 937, similarly to protein level (Figure 7 and Figure 8). The stomatal density of resistant clones was within the parameters cited by different authors for other rubber tree clones (364 stomata·mm^−2^); nevertheless, the mean number of stomata per mm^2^ in our studies was higher for clone RRIM 600 [29,30]. This is probably due to the different counting techniques adopted and to the morphological and environmental factors that can influence stoma formation. A high number of stomata may favor the direct penetration of the fungus into the plant, but the opening period and the location of these stomata must be considered to assure their true role in plant resistance to pathogens.

Depressions in the guard cells may favor the adherence of *Colletrotrichum* spores to the host and, as shown in Figure 7, were more pronounced in RRIM 600 than in resistant clones. Pereira et al. [31], described that penetration of *C. gloeosporioides* in coffee plants was mostly direct and rarely through the stomata, but fungal conidia frequently adhere to the depression at epidermal cells and guard cells.

The obtained results help understand the mechanisms involved in the resistance of rubber tree clones to anthracnose, paving the way for further studies that can contribute to selecting materials resistant to this disease.

## 4. Conclusions

This study found that the accumulation of lignin and lipids was observed more significantly in clones inoculated with *C. tamarillo*. In the case of tannins, they were detected more frequently in conducting vessels and in a smaller amount in the epidermis, differing slightly between inoculated and non-inoculated plants. Arginine was observed most strongly in clones IAC 502 and IAC 507. No starch grains were found in the analyzed samples. The activity of the peroxidase enzyme was higher in resistant clones, mainly in samples with symptoms of anthracnose. The opposite was verified for the protein level and stomatal density, being lower in these clones. The activity of the PAL enzyme did not differ between the samples analyzed.

## Data Availability

The data presented in this study are available on request from the corresponding author. The data are not publicly available due to be part of a bigger project.
